# Essential Oils of Seven Lamiaceae Plants and Their Antioxidant Capacity

**DOI:** 10.3390/molecules26133793

**Published:** 2021-06-22

**Authors:** David Aebisher, Jan Cichonski, Ewa Szpyrka, Sygit Masjonis, Grzegorz Chrzanowski

**Affiliations:** 1 Department of Photomedicine and Physical Chemistry, Institute of Medical Studies, Medical College of Rzeszów University, Warzywna 1A Street, 35-959 Rzeszów, Poland; 2 Department of Biotechnology, Institute of Biology and Biotechnology, University of Rzeszow, 8B Zelwerowicza Street, 35-601 Rzeszow, Poland; janj.cichonski@gmail.com (J.C.); ewaszpyrka@interia.pl (E.S.); sygit@tlen.pl (S.M.)

**Keywords:** antioxidant, phenolic constituents of essential oils, thyme, savory, marjoram, sage, hyssop, DPPH, ABTS

## Abstract

Oxidative stress has been reported as a cause of many diseases like Parkinson′s, Alzheimer′s, cardiovascular disease, and diabetes. Oxidative stress can also lead to cancer formation by promoting tumor development and progression. Antioxidants derived from Lamiaceae plants play an important role in natural medicine, pharmacology, cosmetology, and aromatherapy. Herein, we examine the antioxidative capacity of essential oils from seven aromatic Lamiaceae plants against the synthetic radicals DPPH and ABTS. Among the essential oils analyzed, the most robust scavenging capacities were found in mixtures of volatile compounds from thyme and savory. The scavenging activity of tested EOs against the ABTS radical was clearly higher than activity towards DPPH. Analysis of essential oils with weaker antioxidant activity has shown that volatile compounds from marjoram, sage, and hyssop were more active than EOs from lavender and mint. It can be suggested that the potent antioxidant capacity of thyme (*Thymus vulgaris*) and savory (*Satyreja hortensis*) are related to a high level of phenolic constituents, such as thymol and carvacrol. On the other hand, the elevated antioxidative power of marjoram, sage, and hyssop essential oils may also be due to their terpinene, o-cymene, terpinolene, and terpinen-4-ol constituents. Although non-phenolic components are less active than thymol or carvacrol, they may affect antioxidant capacity synergistically.

## 1. Introduction

Life is a complex process that requires energy to maintain itself. One of the crucial elements necessary for life to exist on Earth is oxygen. Aerobic organisms use this molecule to produce adenosine triphosphate (ATP) via electron transport in the respiratory chain within mitochondria. This metabolic transformation produces oxygen-derived molecules and, among them, reactive oxygen species (ROS), which are comprised of both free radicals, and non-radical (molecular) forms [1,2]. The hydroxyl radical is one of the most potent oxidants and is able to react with lipids, proteins, and nucleic acids. In the absence of a mechanism for the elimination of this radical, excess production of •OH ultimately leads to cell death [3].

Aside from mitochondrial respiration, ROS can also be produced at other endogenous sites such as the endoplasmatic reticulum, peroxisomes, cytosol plasma membrane, extracellular space, or as a result of exogenous stress-inducing sources, such as UV light, gamma radiation, tobacco usage, environmental chemicals, xenobiotics, or pathogens. Reactive oxygen species formed at low or moderate concentrations play an important role in many physiological processes. They perform as secondary messengers in intracellular signaling pathways, gene expression, immune response regulation, and maintaining cellular homeostasis [4]. Reactive oxygen species are involved in the maturation process of cellular structures. Phagocytes can release them as a defense mechanism to destroy invading pathogenic microbes. They take part in the regulation of intracellular signaling cascades in fibroblasts and other nonphagocytic cells. They also participate in a biological system of adaptation to hypoxia, regulation of autophagy, promotion and resolution of inflammation, stem cell differentiation, and longevity [5,6,7]. Organisms regulate the level of ROS to maintain redox homeostasis by deploying antioxidant mechanisms that diminish the content of free radicals and protect against further oxidative bursts. When anti-oxidant protection is interrupted during pathological conditions or stress, excess free radicals lead to deleterious effects and have destructive implications for cells. Excessive levels of ROS may directly damage lipids containing carbon-carbon double bonds such as cholesterol, glycolipids, phospholipids, and polyunsaturated fatty acids. It causes damage to cell membranes, increasing their permeability. Due to the high instability of lipid hydroperoxides, they are further degraded into reactive secondary products such as malondialdehyde (MDA) and conjugated dienes, which are known to be cytotoxic as well as mutagenic [8,9]. Protein oxidation can rapidly contribute to structural modification of cellular proteins and the alteration of their functions. Amino acids are directly modified by ROS via side-chain reactions, especially those containing aromatic and sulfhydryl groups [10]. Oxidative stress has been reported as a reason for many diseases such as Parkinson′s, Alzheimer′s, cardiovascular disease, heart failure, insulin resistance, and diabetes [11,12,13]. Reactive oxygen species can also lead to cancer formation by promoting tumor development and progression. Free radicals produced in cancer cells are released into the tumor microenvironment, which influences angiogenesis and propagation [14]. Reactive oxygen species are also related to aging. The oxidative theory of aging states that cellular senescence and age-related losses of function have a source in the accumulation of oxidative damage to macromolecules over time [15].

Animal and plant organisms are equipped with several antioxidant agents that counteract excessive ROS levels and counterbalance the effect of oxidants. These can be divided into two groups: enzymatic and nonenzymatic. The main enzymatic scavengers include superoxide dismutases (SODs, EC 1.15.1.11), catalase (CAT, EC 1.11.1.6), and glutathione peroxidase (GSH-Px, EC 1.11.1.9). Besides these, other proteins should also be considered as scavengers, such as thioredoxins (TRXs, EC 1.8.4.10) and peroxiredoxins (PRXs, EC 1.11.1.15) [16,17]. To protect against toxic oxygen intermediates, organisms also employ non-enzymatic antioxidants such as vitamins C and E, β-carotene, and glutathione [18,19]. 

Secondary metabolites are defined as valuable natural compounds derived from natural sources and have important biological activities [20]. Plants produce over 200,000 compounds, mostly from specialized metabolite pathways. Due to their chemical structures, polyphenolic compounds and flavonoids are able to diminish the level of reactive oxygen species. Moreover, essential oils (EOs) have antioxidant properties, and the use of EOs as natural antioxidants is a field of growing interest. Synthetic antioxidants, such as BHA and BHT, are now suspected to be potentially harmful to human health [21,22]. Rahimi et al. has reported the efficacy of herbal medicines in inflammatory and oxidant-related diseases [23]. Moreover, Ruberto and Baratta suggest that EOs possess high potential as natural food preservation agents [24].

Studies addressing the antibacterial activity of essential oils has been increasing over the past several years and EOs have been shown to be effective even against multidrug-resistant strains. Antibacterial activity is determined by chemical composition which, for EOs, frequently contain terpenes and oxygenated natural products, such as phenols, alcohols, aldehydes, ketones, and ethers [25,26].

The family Lamiaceae contains many valuable medicinal plants that are rich in EOs and other secondary metabolites. Moreover, they play an important role in natural medicine, pharmacology, cosmetology, and aromatherapy [27,28]. In recent years, there has been an increased interest in the plants from the Lamiaceae family and their application as a source of nutraceuticals. This plant family has a cosmopolitan distribution but are mostly distributed in Mediterranean and Eastern Asiatic regions [29]. Due to the valuable components of essential oils in Lamiaceae plants, they are cultivated in many regions of Europe. However, the origin of plants and environmental conditions may modify the chemical composition of EOs [30].

In the present work, the antioxidant activity of seven EOs obtained from plants belonging to the Lamiaceae family were examined. Essential oils were extracted by hydrodistillation from *Thymus vulgaris*, *Origanum majorana*, *Salvia officinalis*, *Hyssopus officinalis*, *Lavandula angustifolia*, *Mentha x piperita,* and *Satureja hortensis* pharmacopeial feedstock plant material. Antioxidant capacity was tested against two synthetic radicals, 2,2-diphenyl-1-picrylhydrazyl (DPPH) and 2,2′-azino-bis(3-ethylbenzothiazoline-6-sulfonic acid (ABTS).

Then, antioxidant capacity was tested against two synthetic radicals: DPPH and ABTS. Moreover, the composition of the EOs analyzed were examined using gas chromatography coupled mass spectrometry.

## 2. Results and Discussion

Essential oils biosynthesized by plant species are widely distributed in the plant kingdom. In aromatic plants, volatile organic compounds descend from three groups, including phenolic compounds, derivatives of fatty acids, and isoprenoids. The predominant constituents of plant essential oils are isoprenoids (also defined as terpenes or terpenoids) [31]. Due to their toxicity, terpenoid accumulation in plants is generally restricted to specialized secretory structures called glandular trichomes, which are multicellular epidermal hairs in the plants belonging to the Lamiaceae or Asteraceae families [32].

### 2.1. Chemical Composition of the Essential Oils

**Figure 1:**
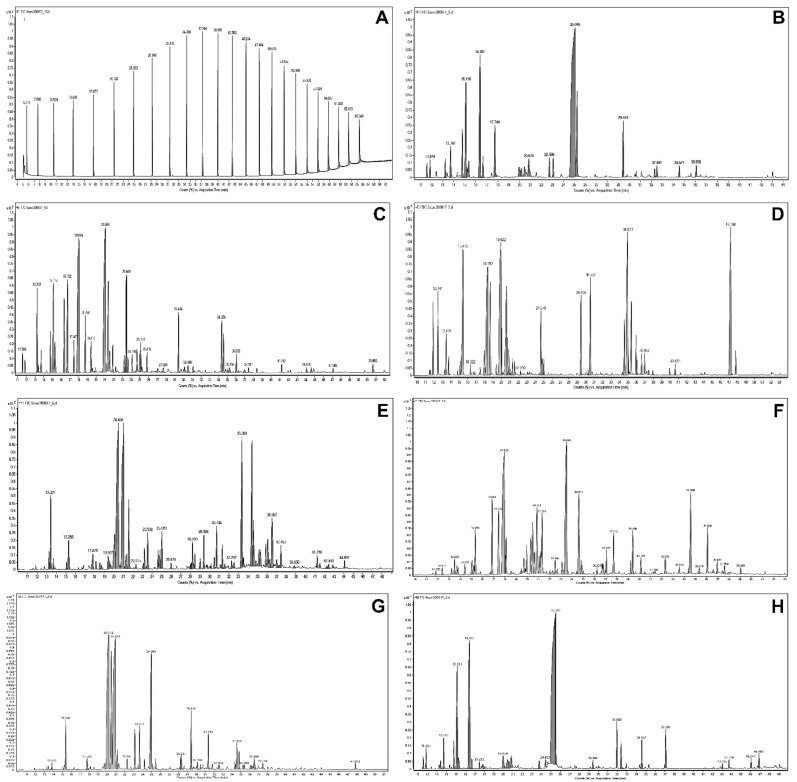
Chromatograms of serrated alkanes and analyzed essential oils; (**A**)—C7-C30 alkanes, (**B**)—*T. vulgaris*, (**C**)—*O. majorana*, (**D**)—*S. officinalis*, (**E**)—*H. officinalis*, (**F**)—*L. augistifolia*, (**G**)—*M. piperita*, (**H**)—*S. hortensis*.

Chromatographic spectra are shown in Figure 1, and yields of essential oils obtained from tested Lamiaceae plants are listed in Table 1. The oil yields ranged between 1.29% for hyssop and 3.95% for savory. We have found that the EO extracted from *Thymus vulgaris* predominantly contained thymol (46.6%), γ-terpinene (11.3%), *p*-cymene (8.1%), and carvacrol (6.4%), at a high level (Table 1). The main components of the EO from *Origanum majorana* were (+)-terpinen-4-ol (24.1%), terpinolene (17.1%), and linalyl acetate (6%). Moreover, α-terpinene, *p*-cymene, *trans*-4-thujanol, and α-terpineol were isolated and detected in similar levels in marjoram essential oils. α-Thujone (13.8%), camphor (13.7%), viridiflorol (13.7%), 1,8-cineole (8.2%), and epimanool (10.9%) were the main components EO from leaves of *Salvia officinalis*. Chromatographic analysis has shown that essential oil from *Hyssopus officinalis* mainly consists of pinocamphone (28.2%) and isopinocamphone (24.5%). 

Moreover, hedycaryol and spathulenol were found in amounts higher than 7.5% in hyssop EO. The EO from the dried flowers of *L. angustifolia* contained mainly linalool (17.8%) and linalyl acetate (15.9%). The content of β-pinene and 1,8-cineole exceeds 7.5 percent slightly. The essential oil from the leaves of *Mentha x piperita* mainly contained menthone (33.6%) and menthol (26.0%). Mint EO included menthyl acetate (12.4%) and menthofuran (11.1%). The predominant component of oil from aerial parts of *Satureja hortensis* was carvacrol. Moreover, a high content of γ-terpinene and *o*-cymene was determined in savory essential oil, 14.9% and 9.1%, respectively.

It should be noted that carvacrol was found only in the essential oils of two Lamiaceae species: *T. vulgaris* and *S. hortensis*. Likewise, thymol, which was found within two essential oils: *T. vulgaris* and *O. majorana*.

We have found slight differences in the chemical composition of essential oils than those reported by other researchers [33,34,35,36]. Moreover, there was a relatively low thymol content in thyme plants (15% lower) than we showed in our earlier work [37]. However, Kowalski and Wawrzykowski [34] found a similar level of thymol in plants of *T. vulgarsis*. Moreover, De Lisi et al. [33] suggest that plants belonging to the *T. vulgaris* biotype are richer in thymol, whereas *Thymus citriodorus* is characterized by geraniol as the major constituent, although, we did not detect geraniol in the analyzed thyme plant. In contrast to Kowalski and Wawrzykowski [34], we report different terpinene and carvacrol contents in thyme. In contrast to their results, we have also found differences in the content of menthone and menthofuran in essential oil from mint and caryophyllene and thujones from sage EO.

Boruga et al. [39] reported that similar studies from Poland, Iran, Spain, and Italy on the differences of thymol, *p*-cymene, and γ-terpinene content in thyme essential oil can be attributed to a large extent to different chemotypes of aromatic plant species.

Results of essential oil component analysis from marjoram, sage, lavender, and mint are similar to our earlier reports [37]. Nurzynska-Wierdak et al. [35] determined significantly higher contents of compounds such as camphene, sabinene, myrcene, and particularly linalool in *O. majorana* essential oil. Moreover, Zawislak [36] found some differences in the composition of salvia EO. Generally, they have detected a higher amount of almost all constituents except for camphor and viridiflorol. In comparison to Baj et al. [40], we did not detect sabinene, and the content of β-pinene and 1,8-cineole were smaller. The amount of hedycaryol and spathulenol significantly exceeded the concentration reported by Baj et al. [40] and Wesołowska et al. [41]. The result most similar to literature data was the composition of essential oil from savory. Mihajilov-Krstev et al. [42] found the same compounds at similar levels as reported in our experiment. An exception was carvacrol content. We report a 17% lower content of oxygenated monoterpene than Mihajilov-Krstev et al. [42].

### 2.2. Antioxidative Activity of the Essential Oils

Regarding the essential oils, various chemotypes have been described within Lamiaceae species describing the main oil components and their biosynthesis [43]. Essential oil constituents which play an important role in natural medicine, pharmacology, cosmetology, and aromatherapy include: (1) monoterpene hydrocarbons, such as myrcene, terpinolene, terpinenes, *p*-cymene; (2) oxygenated monoterpenes: geraniol, lavandulol, terpinen-4-ol, carvacrol, and thymol; (3) oxygenated sesquiterpenes: farnesol and germacrone; as well as derivatives of benzene: eugenol, trans-anethole, and guaiacol [28]. In particular, phenolic compounds and essential oils that contain them have been shown to have strong antioxidant activity [24].

The antioxidant properties of Lamiaceae species have been reported by various authors and often compared to those of other plant materials. Antioxidants react with free radicals by different mechanisms, namely the transfer of hydrogen atom or electron transfer mechanisms. In most mechanisms, these two reactions take place simultaneously, and the antioxidant’s structure and solubility determine the mechanism of the reaction.

Generally, during ABTS assays, there is hydrogen transfer. Single electron transfer takes place during assays based on DPPH reduction [44]. Although the literature reports that DPPH is chiefly attributed to hydrogen transfer reactions, strong hydrogen-bonding solvents such as methanol interfere with the release of hydrogen atoms and thus strongly enhance SET over HAT [45]. 

DPPH (2,2-diphenyl-1-picrylhydrazyl) is a stable, synthetic free radical with an unpaired electron that is delocalized. An additional electron is transferred together with hydrogen. The ABTS assay utilizes a free radical mono-cation of (2,2′-azino-bis 3-ethylbenzothiazoline-6-sulphonic acid) generated by oxidation with potassium persulfate and greenish radical ${\mathrm{ABTS}}^{•+}$ is produced.

**Figure 2:**
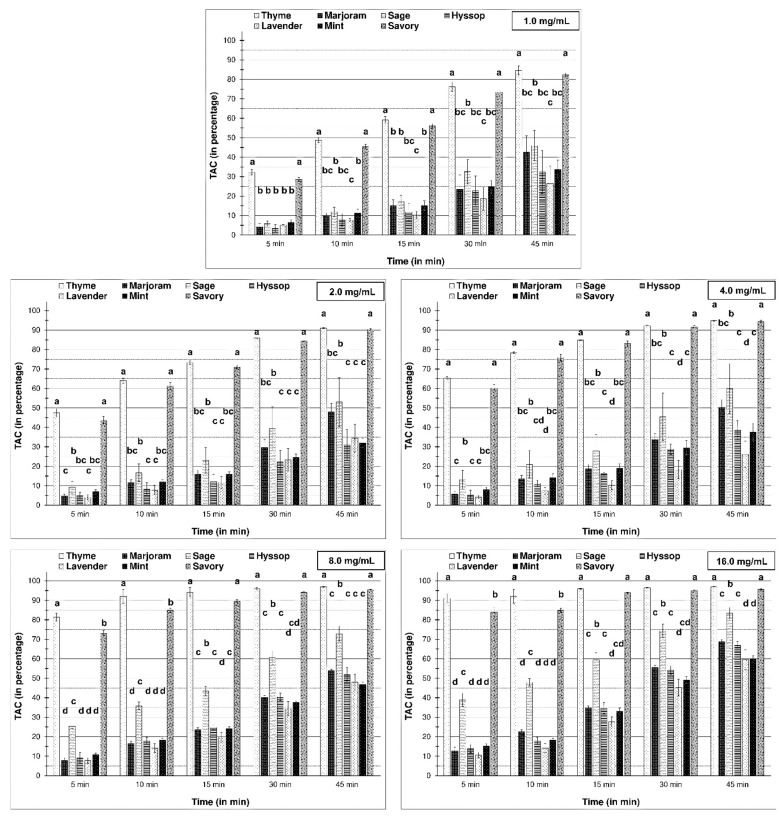
Total antioxidant capacity (as percentage of scavenging) of examined essential oils against DPPH radical; 1.0, 2.0, 4.0, 8.0, 16.0 mg/mL are concentration of essential oils in the reaction mixtures. The bars for each time period marked by different letters are statistically different at *p* < 0.05 (Tukey’s test).

**Figure 3:**
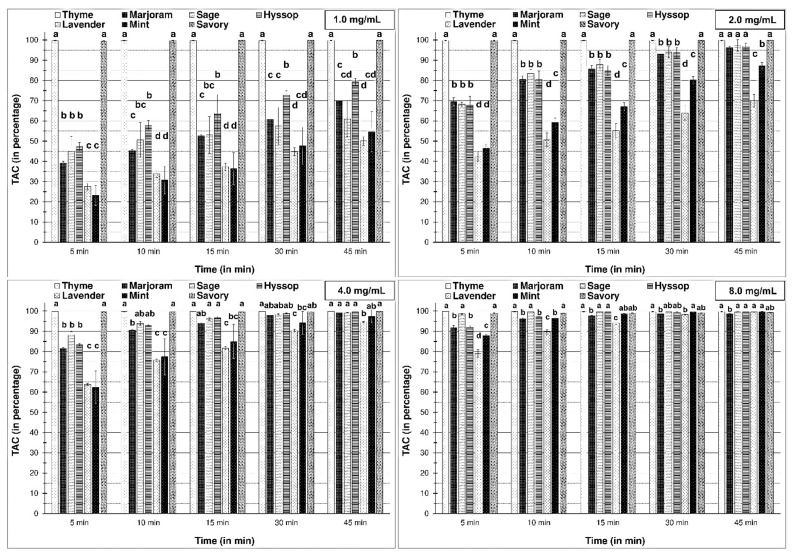
Total antioxidant capacity (in the percentage of scavenging) of examined essential oils against ${\mathrm{ABTS}}^{•+}$ radical; 1.0, 2.0, 4.0, 8.0 mg/mL are concentration of essential oils in the reaction mixtures. The bars for each time period marked by different letters are statistically different at *p* < 0.05 (Tukey’s test).

In vitro antioxidant tests are designed to mimic oxidation-reduction reactions occurring in live biological systems for estimation of the antioxidant potentials of various chemical and biological samples. In this research, the two most widely used assays, DPPH and ABTS tests, were applied to evaluate the antioxidant capacities of essential oils (Figure 2 and Figure 3). The scavenging activity of tested EOs against ABTS radical was clearly higher than towards DPPH. Moreover, Gil et al. [46] found values for the ABTS test were generally significantly higher than for the DPPH assay. Despite this, they should be viewed as a confirmation of the DPPH assay. 

Among the tested essential oils extracted from Lamiaceae species, the most robust scavenging capacity came from mixtures of volatile compounds within thyme and savory. That ability increased gradually over time, achieving the highest level after 45 min-from an initial level of about 30% to over 80% at the end of the experiment (for the lowest thyme EOs concentration). Moreover, comparing the results for different concentrations of EOs suggests that essential oils at the lowest concentration showed, relatively, the highest antioxidant capacity. Primarily, this was seen for essential oils with the highest ability, where *T. vulgaris* EO at concentration 1 mg/mL caused 32% scavenging of DPPH (at 5 min), while the increase in concentration to 25% entailed only a three-fold rising of scavenging capacity.

Analysis of the total antioxidant capacity (TAC) results for the essentials oils with the lowest capacity against DPPH radical showed a significant increase in the activity of sage EO, especially following an increase in concentration.

After analysis with synthetic ${\mathrm{ABTS}}^{•+}$ radical, some similarities to the DPPH assay were found. However, the lofty power of ${\mathrm{ABTS}}^{•+}$ radical scavenging precludes comparing the antioxidant activity of the essential oils that were added at the highest concentrations, particularly at 8 mg/mL. We observed that all essential oils at the highest concentrations completely diminished the level of ${\mathrm{ABTS}}^{•+}$. Moreover, it was exciting to find that the lowest concentration of essential oils from thyme and savory completely scavenged the ${\mathrm{ABTS}}^{•+}$ radicals. In addition, this was observed after 5 min, which was the shortest reaction time.

Analysis of essential oils with weaker antioxidant activity demonstrated that volatile compounds from marjoram, sage, and hyssop were more active than EOs from lavender and mint; this was reported at 3.0% concentration. Moreover, three essential oils (from *O. majorana*, *S. officinalis*, and *H. officinalis*) at a concentration of 2 mg/mL, scavenged ${\mathrm{ABTS}}^{•+}$ radical to the same extent as thyme and savory after 45 min.

The results obtained showing a prolonged DPPH reaction and rapid reaction with ${\mathrm{ABTS}}^{•+}$ radical suggest different reactions than the earlier proposed SET reaction for DPPH radical and HAT reaction for ABTS. However, the application of methanol as a reaction environment indicates that it was a SET reaction for the DPPH test. The question is: Why was a reaction with DPPH radical slower than the ABTS test? Salamone et al. [47] and Salamone et al. [48] explain that despite that the initial reaction of antioxidants with DPPH is quite rapid and occurs according to the electron transfer mechanism, it is in fact much slower than ${\mathrm{ABTS}}^{•+}$ reactions due to decreased access of phenols at the DPPH radical site. Only an increase in the concentration of essential oils containing a high level of phenolic constituents remarkably increased the reaction rate with DPPH radical. This was reported also by Xie and Schaich [49]. Thus, it can be suggested that the potent antioxidant capacity of thyme (*T. vulgaris*) and savory (*S. hortensis*) are related to a high level of phenolic constituents, such as thymol and carvacrol. Ruperto and Barata [24] showed a high antioxidant capacity of thymol and carvacrol against lipid peroxidation. High antioxidative ability is also found in terpinene, o-cymene, terpinolene, and terpinen-4-ol. Moreover, when comparing antioxidative effectiveness, the tested volatile compounds showed significant similarity to the tocopherol, particularly in the high concentration. While most authors and results presented in this work report the high antioxidative properties of thyme essential oils, Dorman et al. [50] demonstrated a low capacity of thyme and high capacity from marjoram EOs and they found a shallow content of phenolic constituents in the thyme essential oil. On the other hand, we have shown the weak antiradical activity of *O. majorana* essential oil since due to elevated contents of antioxidant monoterpenes, such as *p*-cymene and terpinene, terpinolene, and terpinen-4-ol.

**Figure 4:**
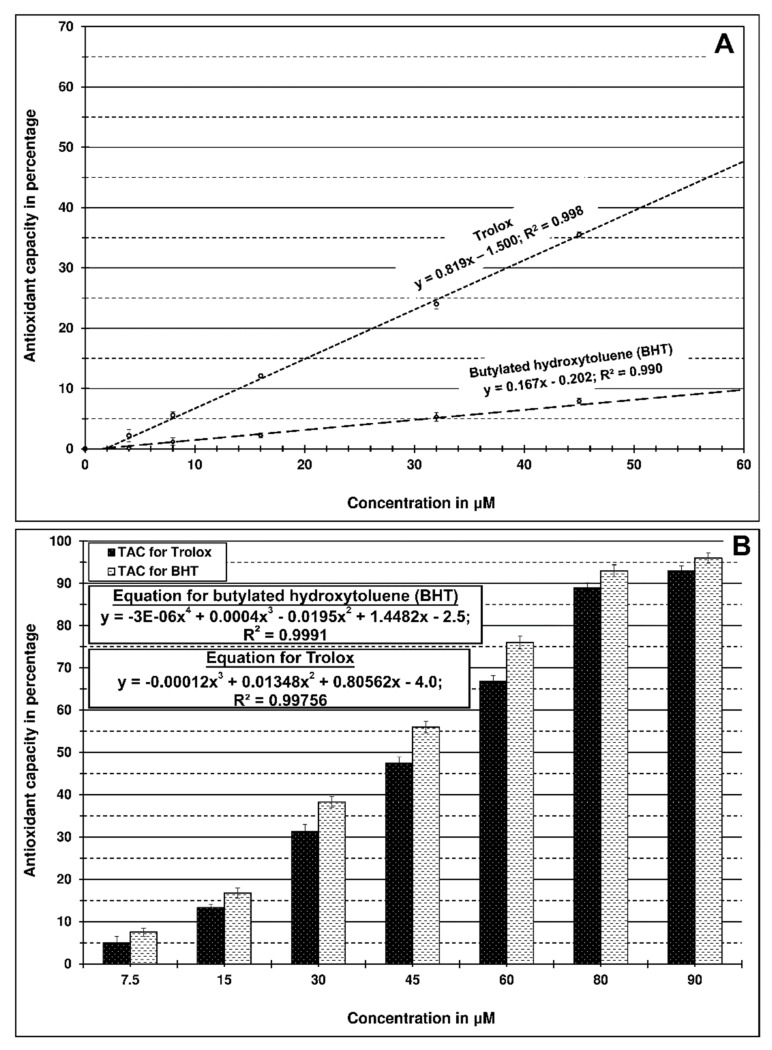
Antioxidative capacity of synthetic trolox and bytylated hydroxytoluene used as positive control; (**A**)—against DPPH radical, (**B**)—against ABTS radical.

Antioxidant activity determination has demonstrated that the synthetic antioxidants Trolox and butylated hydroxytoluene containing a phenolic ring strongly scavenged ABTS compared to the DPPH radical (Figure 4).

These results may suggest some resemblance between the antioxidant activity of essential oils and BHT or Trolox, particularly since the essential oils contain a high level of phenolic constituents. However, it should be emphasized that synthetic compounds used as a positive control occurred at significantly lower concentrations than essential oils.

Mighri et al. [51] studied the antioxidant activity of four types of essential oils from *Artemisia herba-alba*. β-thujone rich oil showed the best inhibition at 12.5%. Nonetheless, the activity of all *Artemisia* oils was significantly lower than the activity of rutin (54.1%) or BHA (89.2%). Thus, these weak activities were attributed to the dominance of non-phenolic compounds in the examined oils. 

## 3. Materials and Methods

### 3.1. Plant Materials

Essential oils were extracted from seven herbs belonging to the Lamiaceae family. The herbs used as a source of essential oils were obtained from commercial herb suppliers. The herbs *Lavandula angustifolia* (*Lavandulae flos*, lot no. 1070.2020), *Mentha x piperita* (*Menthae piperitae folium*, lot no. 1198.2020), *Salvia officinalis* (*Salviae folium*, lot no. 013.2021), *Satureja hortensis* (*Saturejae herbae*, lot no. 016.2021), and *Thymus vulgaris* (*Thymi herba*, lot no. 1124.2020) were purchased from Kawon (Gostyn, Poland). *Hyssopus officinalis* (*Hyssopi herba*, lot no. 1100), *Origanum majorana* (*Majoranae herba*, lot no 1120), were purchased from Flos (Mokrsko, Poland).

### 3.2. Extraction of Essential Oils

Essential oils were extracted from 50 g of the plant samples by hydrodistillation using a Dering-type apparatus for 3 h. The obtained oils were dried over anhydrous sodium sulfate, centrifuged (2000× *g* for 20 min), and stored in an amber bottle at 4 °C until analysis. The extraction was carried out in triplicate for yield calculation. The yield was expressed as mL of essential oil/100 g of dry material (Table 1). For a total antioxidant capacity determination, EOs were dissolved with ethanol to a 50% (*w*/*v*) concentration (Stock solution). The examination was performed immediately following preparation of stock solutions.

### 3.3. Analysis of Essential Oil Composition 

Chromatographic analysis was performed on a 7890A gas chromatograph (Agilent Technologies, Palo Alto, CA, USA) equipped with a mass detector, model 7000 (GC-MS/MSQqQ), working in electron impact mode. The extracts were separated on a HP-5MS Ultra Inert column (30 m × 0.25 mm ID × 0.25 μm). 

Samples of essential oils were diluted 100-fold with with n-hexane (purity–for residue analysis, Witko, Lodz, Poland). The sample injection volume was 5µL, and split injection was used (split ratio 1:20) at split-flow 10 mL/min. Helium (5.0 purity) was used as the carrier gas at a flow rate of 0.5 mL/min. The analyses were carried out in the programmed mode with a temperature gradient of 50–300 °C at 4 °C/min. The mass spectrometer was used in electron impact (EI) ionization mode (70 eV). Ions were monitored in full scan mode in the range of 20–400 *m*/*z*. The software Mass Hunter version B.07.06 was used for the acquisition and processing of the mass data. The identification of individual compounds was based on the calculated retention indices (RI) and on comparisons of obtained mass spectra with those of reference compounds that are available in the NIST (National Institute of Standards and Technology, Gaithersburg, MD, USA) library, MS data from the literature [4,38,52] and our library databases. The retention indices were determined versus C7–C30 alkanes (certified reference material #49451-U, Sigma-Aldrich, Co. LLC, St. Louis, MO, USA). Quantitative analysis of the essential oil components was performed in the same operating conditions using a flame ionization detector (GC-FID).

### 3.4. Total Antioxidant Capacity (TAC) 

The antioxidant capacity of samples was evaluated using both a DPPH assay and an ABTS assay. The Infinite 200 PRO multimode plate reader (Tecan Group Ltd., Männedorf, Switzerland) was used in both assays. The free radical-scavenging capacity was calculated using Equation (1), and expressed as a percentage ± standard deviation.

(1) $$\mathrm{Radical}\;\mathrm{scavenging}\;\left(\%\right)=\frac{\left(A_{\left(\mathrm{DPPH}/{\mathrm{ABTS}}^{+}\right)}-A_{\left(\mathrm{sample}\right)}\right)}{A_{\left(\mathrm{DPPH}/{\mathrm{ABTS}}^{+}\right)}}\times 100$$

The DPPH assay was carried out according to the procedures used by Brand-Williams et al. with slight modifications [53]. A solution of DPPH (2,2-diphenyl-1-picrylhydrazyl (Sigma-Aldrich, Co. LLC, St. Louis, MO, USA), 0.1 mM in MeOH, was used with an absorbance of 0.95 (± 0.03) at 517 nm. Appropriate concentrations of essential oil samples were prepared from a 50% stock solution and 10 μL of each sample was added to 140 μL of DPPH solution in wells of a 96-well plate. The final concentrations of essential oils in the reaction mixtures were as follows: 1.0, 2.0, 4.0, 8.0, and 16 mg/mL. Absorption was measured after 5, 10, 15, 30, and 45 min from the sample addition at 517 nm.

The ABTS assay was performed based on a procedure reported by Re et al. with slight modifications [54]. ABTS (2,2′-azino-di-(3-ethylbenzthiazoline sulfonic acid) (Sigma-Aldrich, Co. LLC, St. Louis, MO, USA) was dissolved in water for a 7.5 mM solution. ABTS radical cation (${\mathrm{ABTS}}^{•+}$) was generated by reacting 7.5 mM ABTS with 2.5 mM potassium persulfate (final concentration). The mixture was allowed to stand in the dark at room temperature for 16 h before use. The ${\mathrm{ABTS}}^{•+}$ stock solution was diluted with methanol to an absorbance of 0.90 (±0.02) at 734 nm. The diluted ABTS solution (140 μL) was mixed with 10 μL of the sample prepared from a 50% stock solution of essential oil. The final concentrations of essential oils in the reaction mixtures were as follows: 1.0, 2.0, 4.0, 8.0, and 16 mg/mL. The absorption was measured after 5, 10, 15, 30, and 45 min from the sample addition at 734 nm.

Trolox and butylated hydroxytoluene (BHT) (Sigma-Aldrich, Co. LLC, St. Louis, MO, USA) were used as a positive control.

### 3.5. Statistical Analysis

All determinations were conducted in triplicate, and the results were expressed as mean ± standard deviation (SD). Data were analyzed by one-way analysis of variance (ANOVA) using Statistica, v. 13.3 [55]. Significances of differences were calculated using Tukey’s multiple range test (*p* ≤ 0.05).

## 4. Conclusions

The biological activity of natural compound mixtures depends on the chemical composition and nature of constituents. From different chemical structures, phenolic constituents of essential oils play a relevant role against reactive oxygen species. On the other hand, although non-phenolic components are less active than thymol or carvacrol, they may synergistically affect the antioxidant capacity of phenolic constituents of essential oils. Moreover, phenolic compounds in high concentration may affect prooxidative properties [56]. Marino et al. [57] reported that thymol and carvacrol are the main components of Lamiaceae species responsible for their antimicrobial properties. Moreover, they interfere with cellular metabolism after penetrating the cell and cause antibacterial activity by permeabilizing and depolarizing the cytoplasmic membrane [58]. Carvacrol also inhibits the production of microbial toxins and reduces the biofilm formation of uropathogenic *Escherichia coli* [59]. However, differences in the amount of phenol within essential oils may affect their antimicrobial activity. Even a higher level of other constituents, such as linalool and linalyl-acetate, did not increase antimicrobial potential. They likely modify the antioxidative activity of phenols synergistically [58,60]. Thus, further experiments are needed to elucidate the role of essential oils from Lamiaceae plants as antimicrobial agents and their mechanism of action on different bacteria.

## Figures and Tables

**Table 1:** Chemical constituents of the essential oils of seven Lamiaceae species.

Major Chemical Compounds	*RI* ^a^	*RI* ^b^	*Thymus Vulgaris (%)*	*Origanum Majorana (%)*	*Salvia* *officinalis (%)*	*Hyssopus officinalis (%)*	*Lavandula angustifolia (%)*	*Mentha* *x piperita (%)*	*Satureja hortensis (%)*
α-Thujene	930	929	0.7	-	-	-	-	-	0.6
α-Pinene	938	937	0.9	-	2.2	-	-	-	1.0
Camphene	955	952	-	-	3.0	-	-	-	-
Sabinene	979	974	-	3.8	-	-	2.8	-	-
β-Pinene	983	979	-	-	1.3	3.1	7.4	-	-
β-Myrcene	992	991	1.6	0.9	-	3.7	3.6	-	1.6
2-Carene	1021	1001	2.9	-	-	-	-	-	-
α-Terpinene	1022	1017	-	5.7	-	-	-	-	1.3
o-Cymene	1031	1022	-	-	-	-	-	-	9.1
*p*-Cymene	1030	1025	8.1	5.3	-	-	-	-	-
β-Phellandrene	1035	1031	-	-	-	1.8	-	-	-
1,8-Cineole	1039	1032	-	-	8.2	0.8	8.2	2.7	-
γ-Terpinene	1065	1060	11.3	3.6	-	-	-	-	14.9
*trans*-4-Thujanol	1074	1070	-	5.1	-	-	-	-	-
Linalool oxide	1078	1074	-	-	-	-	4.4	-	-
*trans*-Linalool oxide (furanoid)	1093	1086	-	-	-	-	3.7	-	-
Terpinolene	1094	1089	-	17.1	-	-	-	-	-
Linalool	1102	1099	3.4	-	-	-	17.8	-	-
α-Thujone	1118	1103	-	-	13.8	-	-	-	-
β-Thujone	1127	1114	-	-	4.7	-	-	-	-
Camphor	1161	1145	-	-	13.7	-	-	-	-
Menthone	1156	1154	-	-	-	-	-	33.6	-
Menthofuran	1165	1164	-	-	-	-	-	11.1	-
Pinocamphone	1177	1160	-	-	-	28.2	-	-	-
Borneol	1179	1167	-	-	6.3	-	3.3	-	-
Menthol	1181	1169	-	-	-	-	1.3	26.0	-
Isopinocamphone	1191	1173	-	-	-	24.5	-	-	-
(+)-Terpinen-4-ol	1186	1177	2.1	24.1	1.1	-	-	-	-
(−)-Terpinen-4-ol	1187	1182	-	-	-	-	4.2	-	-
α-Terpineol	1202	1189	-	5.8	-	-	3.8	-	-
(−)-Myrtenol	1207	1213	-	-	-	3.5	-	-	-
Benzene 2-methoxy-4-methyl-1-(1-methylethyl)	1239	1235	1.2	-	-	-	-	-	-
γ-Terpineol acetate	1261	1256	-	3.6	-	-	-	-	-
Linalyl acetate	1260	1257	-	7.6	-	-	15.2	-	-
Lavandulol acetate	1293	1270	-	-	-	-	5.9	-	-
Borneol acetate	1295	1284	-	-	2.5	-	-	-	-
Thymol	1306	1291	46.6	1.4	-	-	-	-	-
Menthyl acetate	1303	1295	-	-	-	-	-	12.4	-
Carvacrol	1311	1299	6.4	-	-	-	-	-	50.8
Geranyl acetate	1385	1382	-	-	-	-	2.2	-	-
Caryophyllene	1438	1454	3.6	2.7	6.7	3.2	2.3	3.5	1.9
Germacrene D	1499	1481	-	-	-	-	-	2.0	-
β-Bisabolene	1519	1509	-	-	-	-	-	-	2.5
Myristicin	1533	1519	-	-	-	-	-	-	1.8
Hedycaryol	1567	1559	-	-	-	7.8	-	-	-
Spathulenol	1599	1576	-	2.5	-	7.6	-	-	-
Caryophyllene oxide	1605	1581	3.7	2.0	2.1	2.1	5.6	1.6	1.8
Viridiflorol	1620	1591	-	-	13.7	-	-	-	-
δ-Cadinol	1658	1640	0.7	-	-	-	2.5	-	-
β-Eudesmol	1674	1649	-	-	-	4.1	-	-	-
α-Eudesmol	1677	1653	-	-	-	3.5	-	-	-
α-Cadinol	1674	1653	-	-	-	-	-	1.1	-
Apiol	1694	1682	-	-	-	-	-	-	2.6
2-Pentadecanone. 6.10.14-trimethyl	1848	1844	-	0.3	-	-	-	-	-
Epimanool	2091	2056	-	-	10.9	-	-	-	-
Phytol	2118	2114	-	-	-	-	-	0.4	-
**Sum**			**93.2**	**91.5**	**90.2**	**93.9**	**94.2**	**94.4**	**89.9**
Oil yield (%, *v*/*w*)			**1.89**	**1.43**	**1.52**	**1.29**	**2.79**	**1.86**	**3.95**
Density (g cm^−3^)			**0.960**	**0.995**	**0.857**	**0.997**	**0.846**	**1.047**	**0.752**

^a^ Retention index calculated; ^b^ Retention index from NIST Database and Adams [38].

## Data Availability

Not applicable.

## Sample Availability

Samples are not available.
